# Dissociation of a Medial Pivot Polyethylene in a Kinematically Aligned Total Knee Arthroplasty

**DOI:** 10.7759/cureus.21813

**Published:** 2022-02-01

**Authors:** Naga Cheppalli, Bryce Clinger, Nicholas W Brady, William Skelly

**Affiliations:** 1 Orthopedic Surgery, Raymond G. Murphy Veterans Affairs Medical Center, Albuquerque, USA; 2 Orthopedic Surgery, University of New Mexico School of Medicine, Albuquerque, USA; 3 Orthopedic Surgery, University of New Mexico (UNM) Hospital, Albuquerque, USA

**Keywords:** kinematically, knee joints, total knee, kinematically aligned total knee arthroplasty, medial pivot polyethylene

## Abstract

An 81-year-old male patient who underwent a Medacta GMK sphere kinematically aligned (KA) total knee arthroplasty (TKA) for end-stage knee osteoarthritis presented with a dislocated medial pivot (MP) tibia polyethylene (PE) insert on routine six-week postoperative x-rays. The patient presented asymptomatic with a normal range of motion. Dissociation of a fixed-bearing (FB) PE implant is an uncommon complication after TKA. There are only a few cases reported in the literature. We report for the first time a case of non-traumatic dissociation of MP PE from the tibial baseplate in a KA TKA in an asymptomatic patient but identified on routine postoperative radiographs.

## Introduction

Kinematically aligned (KA) total knee arthroplasty (TKA) is aimed to restore normal knee function by aligning the distal and posterior femoral joint line according to the functional transverse axes and joint line of the tibial to that of its normal or pre arthritic status [1]. Medial pivot (MP) design in TKA is designed to provide near-normal knee kinematics with a stable point of contact on both the medial and lateral side to allow posterior translation of the femoral condyle during knee flexion [2]. The potential changes in stresses across the joint with this new design and technique are yet to be investigated.

## Case presentation

**Figure 1:**
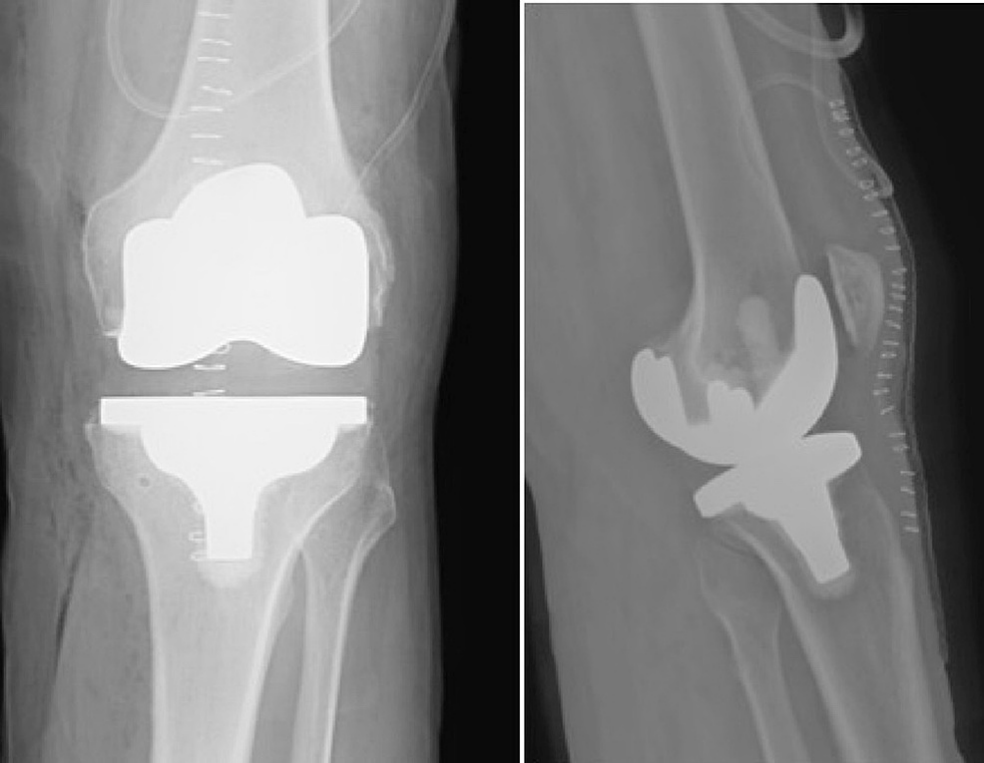
Anteroposterior (left) and lateral (right) radiographs of the immediate postoperative primary total knee arthroplasty.

We report an 81-year-old male (BMI 28.32) who underwent a primary cemented KA TKA using the GMK Sphere (Medacta International AG, Castel San Pietro, Switzerland) (femur 3, tibia 3, and posterior stabilized polyethylene (PE) 11, and a resurfaced patella) through a medial parapatellar approach. The PE was inserted as per the technique guide and securely engaged within the locking mechanism. Intraoperatively the knee was very well balanced, with a range of motion (ROM)of 0-130 degrees (Figure 1).

**Figure 2:**
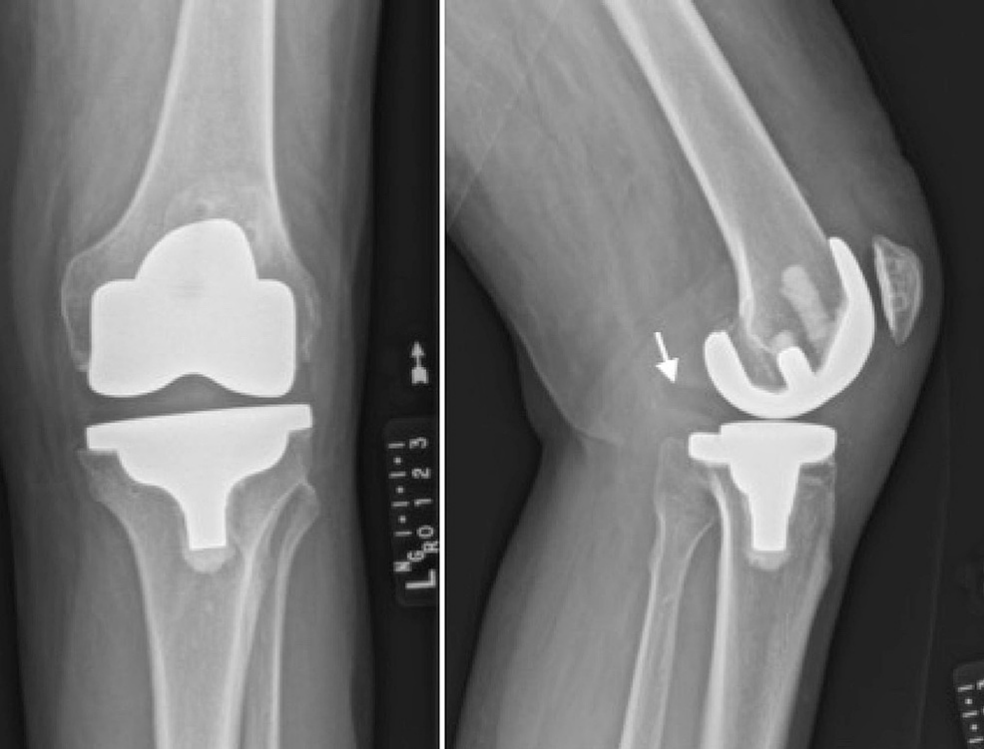
Anteroposterior (left) and lateral (right) radiographs obtained at the 6-week postoperative visit showing posterior dislocation of the tibial polyethylene with asymmetrical joint space.

At two weeks, his postoperative visit was uneventful with a well-healed surgical incision and ROM 0-110 degrees. At the routine six-week follow-up, posterior dissociation of the PE was seen on radiographs. There was no trauma or unusual activity reported by the patient. His ROM was painless from 0 to 125 degrees (Figure 2).

**Figure 3:**
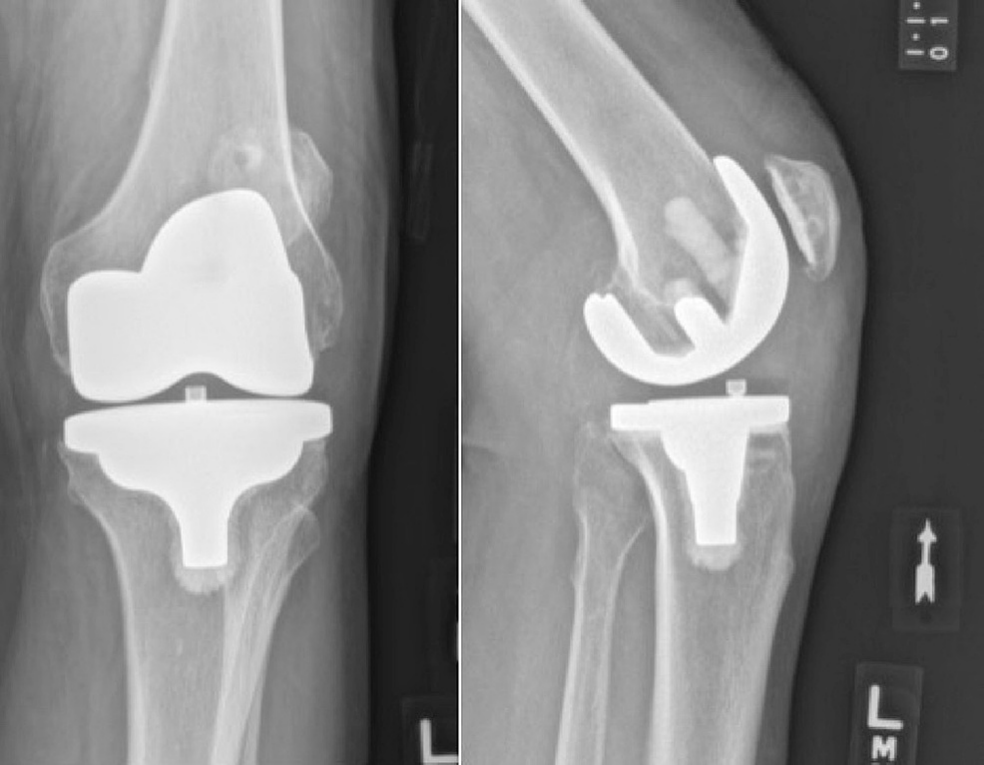
Anteroposterior (left) and lateral (right) radiographs obtained following the revision surgery with polyethylene swap and placement of the central tibial screw.

Plans were made for revision surgery and intraoperatively it was noted that the posterior lateral portion of the PE disengaged the peripheral locking mechanism. No damage to the locking mechanism of the tibial tray was noted. A similar-sized (11 mm) PE was replaced and secured with an optional central screw. At the most recent routine visit (12 months follow-up), the patient was pain-free with 0-120 degrees ROM and had no radiologic signs of PE dissociation (Figure 3).

## Discussion

Dissociation of the PE from a fixed-bearing tibial tray is uncommon. Undiagnosed, it can result in a spin-out and potentially damage ligamentous and neurovascular structures, patellar tendon, and tibial locking mechanism [3,4]. After a literature review, around 20 cases of dissociation of fixed-bearing PE after primary TKA have been documented. No case of dissociated fixed-bearing poly was reported with MP design in KA TKA. Most dissociations happened after the posterior stabilized (PS) TKA [5,6] and a few after cruciate-retaining TKA [7,8]. After a careful review of the existing literature, we can classify dissociation causes as technique-related, implant-related, and patient-related.

Technique-related causes

First, improper placement of the PE during insertion due to inadequate exposure is considered to be a common cause of failure [4]. Second, inadequate soft tissue balance in the sagittal and coronal planes can result in abnormal stresses on locking mechanisms. Tight flexion space can cause the PE to fail at the anterior tabs [9]. Furthermore, if flexion space is loose, abnormal anterior translation of the femoral component can result in posterior tabs failing from locking groves. Third, unusual varus-valgus laxity causes abnormal loading with flexion and adduction, which can cause the failure of the locking mechanism [10]. Fourth, unresected posterior osteophytes from the femur can impinge the posterior aspect of the PE and cause anterior lifting of the poly [11].

Implant-related causes

In PS TKA, with increasing flexion angle, the cam mechanism engages driving the femoral-tibial contact more posteriorly. This can lead to abnormal contact pressure in the posterior half of the PE and can dissociate the locking mechanism anteriorly [5,12]. In CR TKA, tight PCL or inadequate slope can cause abnormal pressure in the posterior half of the poly and result in the anterior tabs failing [13].

Here, we describe the first case in the literature with MP design with a failed locking mechanism posterolaterally. The MP design has a ball and socket configuration on the medial side and a convex configuration on the lateral side in an attempt to replicate native kinematics. However, with this design, it is believed that there is more anterior to posterior translation in the lateral compartment compared to traditional designs (PS and CR). The increased translation in the lateral compartment can increase the forces in the peripheral locking mechanisms. In this case, the abnormally increased pressure on the anterior portion of the lateral compartment poly component caused the posterior lateral dovetail locking mechanism to fail, resulting in dislocation of the PE implant posteriorly.

**Figure 4:**
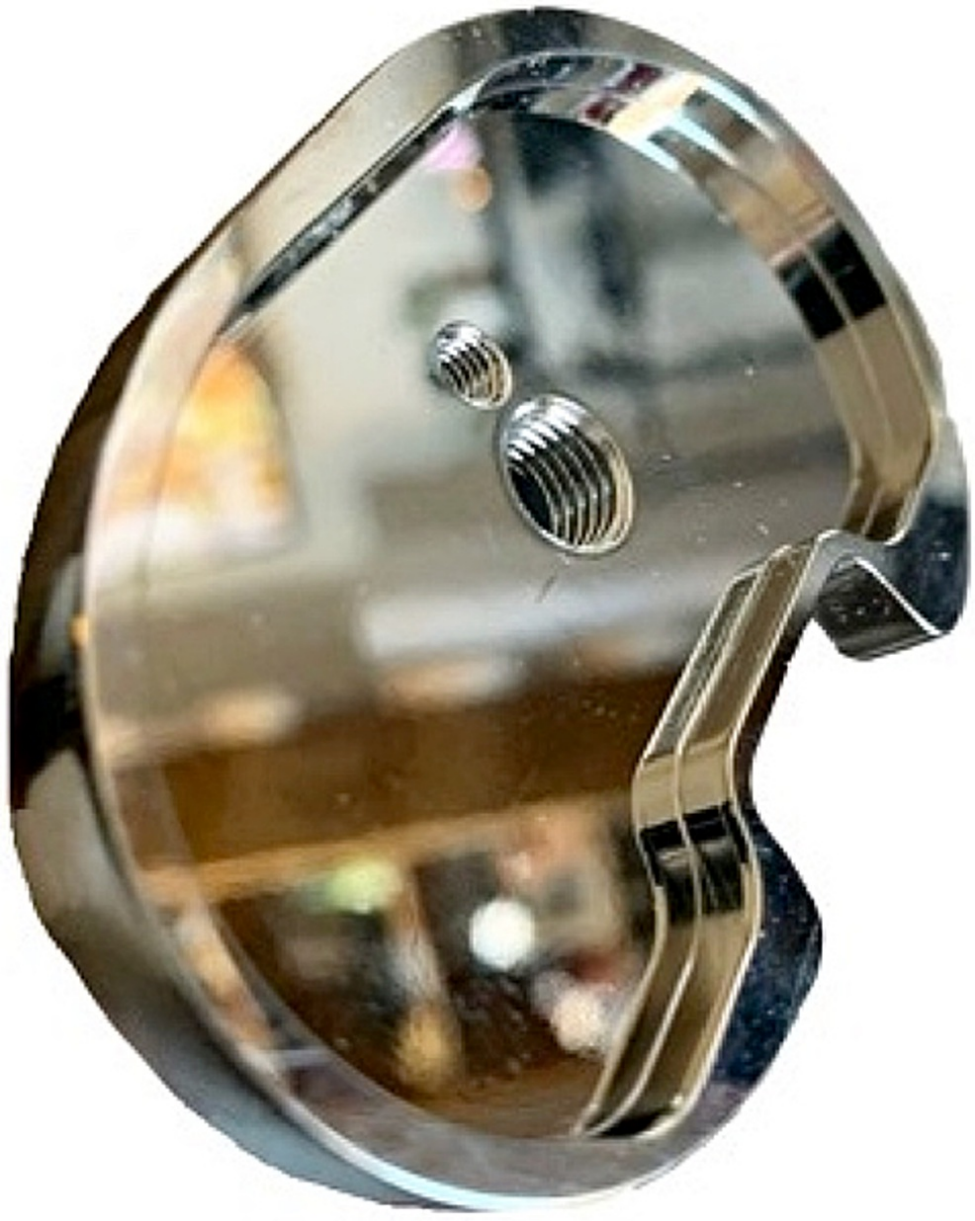
Photo of the Medacta tibial base tray showing the peripheral locking mechanism with the optional central screw hole.

A locking mechanism between the PE and tibial tray can be categorized as linear, peripheral, or central locking. Linear locking mechanisms use the tongue in a groove (Stryker Traithalon, Depuy Attune) with tracks that run on either anterior to posterior and medial to lateral. They can also sometimes have an additional locking pin to prevent anterior lift-off (Biomet Vanguard, Stryker Triathlon). Peripheral capture mechanisms (Smith and Nephew Genesis I and II and Depuy Sigma, Arthrex) have a snap-fit with beveled edges along either the entire periphery or segment of the periphery. Finally, central mechanisms use a mushroom-shaped pin with a peripheral flange for rotational stability or a central locking screw (Medacta). The Medacta tibial base plate has a peripheral locking mechanism and has a central screw that can lock the PE to the tibial baseplate (Figure 4). This central screw is described in the technique guide as an optional step for additional security but is not required as the peripheral capture locking mechanism can suffice.

Patient-related factors

Laxity of soft tissues with atypical translations [7], high BMI [12], repetitive pounding activities [14], failed extensor mechanism [15] are some of the described causes for dissociated fixed bearing PE.

## Conclusions

This is the first case reported with a dissociated PE in a fixed-bearing MP design in KA TKA. Even though the reason for the failed locking mechanism is unclear, we believe the abnormal anterior translation of the lateral compartment and the first-generation peripheral locking mechanism are the reasons for dissociation. However, technique-related causes like improper seating and improperly balanced ligaments are very subjective and could not be ruled out. This case also highlights the importance of regular follow-up and careful clinical and radiological evaluation, especially when adapting to newer techniques and technology, to avoid devastating complications. 

## References

[1] Schiraldi M, Bonzanini G, Chirillo D, de Tullio V (2016). Mechanical and kinematic alignment in total knee arthroplasty. Ann Transl Med.

[2] Sabatini L, Risitano S, Parisi G, Tosto F, Indelli PF, Atzori F, Massè A (2018). Medial pivot in total knee arthroplasty: literature review and our first experience. Clin Med Insights Arthritis Musculoskelet Disord.

[3] Lanting BA, McCalden RW, Naudie DD (2011). Dislocated polyethylene inserts in fixed-bearing total knee arthroplasty. J Arthroplasty.

[4] In Y, Sur YJ, Won HY, Moon YS (2011). Recurrent dissociation of the tibial insert after mini-subvastus posterior-stabilized total knee arthroplasty: a case report. Knee.

[5] Hepinstall MS, Rodriguez JA (2011). Polyethylene subluxation: a radiographic sign of locking mechanism failure after modular total knee arthroplasty. J Arthroplasty.

[6] Rapuri VR, Clarke HD, Spangehl MJ, Beauchamp CP (2011). Five cases of failure of the tibial polyethylene insert locking mechanism in one design of constrained knee arthroplasty. J Arthroplasty.

[7] Poulter RJ, Ashworth MJ (2005). A case of dissociation of polyethylene from its metal baseplate in a "one piece" compression-moulded AGC tibial component. Knee.

[8] Hedlundh U, Andersson M, Enskog L, Gedin P (2000). Traumatic late dissociation of the polyethylene articulating surface in a total knee arthroplasty--a case report. Acta Orthop Scand.

[9] Davis PF, Bocell JR, Tullos HS (1991). Dissociation of the tibial component in total knee replacements. Clin Orthop Relat Res.

[10] Bonorino JFA, Slullitel PA, Kido GR, Bongiovanni S, Vestri R, Carbó L (2015). Traumatic dislodgement of tibial polyethylene insert after a high-flex posterior-stabilized total knee replacement. Case Rep Orthop.

[11] Rutten SG, Janssen RP (2009). Spontaneous late dislocation of the high flexion tibial insert after Genesis II total knee arthroplasty. A case report. Knee.

[12] Voskuijl T, Nijenhuis TA, Van Hellemondt GG, Goosen JHM (2015). Insert dissociation after fixed bearing PS constrained Genesis II total knee arthroplasty. A case series of nine patients. Acta Orthop Belg.

[13] Waelchli B, Romero J (2001). Dislocation of the polyethylene inlay due to anterior tibial slope in revision total knee arthroplasty. Knee Surg Sports Traumatol Arthrosc.

[14] Anderson JA, MacDessi SJ, Valle AGD (2007). Spontaneous, recurrent dislodgment of the polyethylene tibial insert after total knee arthroplasty. A case report. J Bone Joint Surg Am.

[15] Ries MD (2004). Dissociation of an ultra-high molecular weight polyethylene insert from the tibial baseplate after total knee arthroplasty. A case report. J Bone Joint Surg Am.

